# AN EDUCATIONAL INTERVENTION FOR UNDERGRADUATE STUDENTS TO PROMOTE AWARENESS OF END-OF-LIFE OPTIONS

**DOI:** 10.1093/geroni/igad104.0372

**Published:** 2023-12-21

**Authors:** Kelly O’Sullivan, Autumn Decker, Raven Weaver, Cory Bolkan

**Affiliations:** Washington State University, Pullman, Washington, United States; Washington State University, Pullman, Washington, United States; Washington State University, Pullman, Washington, United States; Washington State University Vancouver, Vancouver, Washington, United States

## Abstract

Most people, of all ages, do not engage in advance care planning or know about available end-of-life (EOL) choices, despite the personal and societal benefits in an aging society, highlighting the need for educational intervention and outreach. We implemented an online educational intervention to explore accurate understanding of medical-aid-in-dying (MAID; available in ten states) among college students (n=415; Mage=19.6±4.4 years). Pre-intervention, 66% of participants had heard of MAID and 63% supported or strongly supported MAID. Participants were randomized to three conditions: control, testimonial narrative, or testimonial video explaining reasons why individuals pursued MAID (there were no significant differences in opinion by condition). Post-intervention, those in an intervention group were significantly more likely be more supportive of MAID legislation (p=.005); there were no significant differences between narrative or video conditions. Content analysis of open-ended responses revealed the importance of bodily autonomy and having a choice in health care. Participants who expressed neutral or conflicted perceptions wanted to learn more about the law and were concerned about individuals’ mental health when considering MAID. Negative responses reflected concerns about abusing the law and religious-based concerns. More accurate information about MAID and its safeguards is needed. In particular, the undecided group could benefit from selective interventions to improve understanding of MAID and other EOL care options. Future work should focus on education and outreach to individuals, families, and providers about aging and mortality issues to ensure more equitable access to quality EOL services and supports for people across the life span.

